# A case report of pregnancy complicated by diffuse alveolar hemorrhage: An unexpected link to untreated Graves' disease

**DOI:** 10.1016/j.rmcr.2026.102471

**Published:** 2026-07-16

**Authors:** Sara Soltanmohammadi, Hesam Amini

**Affiliations:** a Thoracic Research Center, Imam Khomeini Hospital Complex, Tehran University of Medical Sciences, Tehran, Iran; b Department of Thoracic Surgery, Imam Khomeini Hospital Complex, Tehran University of Medical Sciences, Tehran, Iran

**Keywords:** Diffuse alveolar hemorrhage (DAH), Graves' disease, Hyperthyroidism, Pregnancy, Hemoptysis

## Abstract

**Background:**

Diffuse Alveolar Hemorrhage (DAH) is a life-threatening condition characterized by widespread bleeding within the alveoli, often resulting from pulmonary capillaritis, bland pulmonary hemorrhage, or diffuse alveolar damage. It presents with hemoptysis, dyspnea, hypoxemia, and anemia, and can lead to respiratory failure. This case report highlights a rare instance of DAH in a pregnant woman with untreated Graves' disease, emphasizing the importance of considering uncommon etiologies in the absence of typical triggers.

**Case presentation:**

A 36-year-old pregnant woman at 37 weeks of gestation presented with hemoptysis and dyspnea. Initial evaluations, including pulmonary CT angiography, ruled out pulmonary embolism but revealed bilateral diffuse alveolar involvement. Bronchoscopy confirmed DAH, with serologic tests indicating hyperthyroidism due to Graves' disease. The patient was treated with pulse methylprednisolone and later methimazole, leading to symptom resolution. She delivered a healthy baby at 40 weeks via uncomplicated vaginal delivery.

**Discussion:**

DAH during pregnancy is rare, and its association with untreated Graves' disease is exceptionally uncommon. In this case, untreated Graves' disease may have contributed to left ventricular diastolic dysfunction, exerting shear stress on pulmonary vasculature and potentially resulting in DAH. The absence of coagulopathy, environmental exposures, or autoantibodies supported this conclusion. This case underscores the need to consider thyroid disorders in the differential diagnosis of bland pulmonary hemorrhage, particularly when other causes are excluded. Early identification and management of underlying causes can significantly improve maternal and fetal outcomes. This report highlights the importance of exploring rare etiologies in cases of DAH, especially in pregnant patients, to ensure timely and effective intervention.

## 1 Background

Diffuse Alveolar Hemorrhage is a life-threatening condition characterized by widespread bleeding within the alveoli, originating from the pulmonary circulation (alveolar capillaries, arterioles, and venules) [1]. Clinically, DAH presents with hemoptysis, dyspnea, hypoxemia, anemia, and diffuse pulmonary infiltrates. In severe cases, it can lead to hypoxic respiratory failure and death [2]. DAH has various etiologies, and identifying the underlying cause is crucial for determining the appropriate treatment plan. Generally, the causes of DAH can be classified into three main categories: pulmonary capillaritis, bland pulmonary hemorrhage, and diffuse alveolar damage [3]. Pulmonary capillaritis is the most common cause, accounting for approximately 50% of cases [2]. In this condition, intense neutrophil infiltration of the pulmonary vascular walls leads to vascular inflammation, necrosis, and hemorrhage [4]. In bland pulmonary hemorrhage, there is no inflammation, necrosis, or structural changes in the lung. Causes of bland hemorrhage include pulmonary hemorrhage due to anticoagulant use, thrombocytopenia, idiopathic pulmonary hemosiderosis, and mitral stenosis [5]. Diffuse alveolar damage (DAD) involves alveolar edema and the formation of hyaline membranes in the alveolar walls, with causes such as ARDS due to infection, drugs, connective tissue diseases, radiation therapy, and pulmonary infarction [5].

Diagnosis is based on history, physical examination, imaging findings, and serial bronchoalveolar lavage (BAL) during bronchoscopy. Bronchoscopy also helps rule out infectious causes [1].

Drug-induced DAH often results from pulmonary capillaritis. Drugs such as propylthiouracil, diphenylhydantoin, penicillin, all-trans-retinoic acid, chemotherapeutic agents, sulfasalazine, hydralazine, carbimazole, leukotriene antagonists, and mitomycin are known to cause DAH through autoimmune mechanisms [6]. Although DAH associated with propylthiouracil, used for hyperthyroidism treatment, is a well-known phenomenon, the occurrence of DAH in the context of primary hyperthyroidism is extremely rare, with only two cases reported to date. This report describes a case of DAH in the context of Graves' disease in a pregnant woman. This case report is prepared in accordance with the CARE guidelines.

## 2 Case presentation

A 36-year-old pregnant woman at 37 weeks and 4 days of gestation presented to the emergency department with moderate hemoptysis that had started 2 h prior. The patient also complained of sudden dyspnea. She denied fever, chills, chest pain, recent immobilization, or any obstetric complications during the current pregnancy. She had no miscarriage history. Throughout her pregnancy, she had not sought medical care and had not received standard prenatal care. On examination, the patient was alert, without respiratory distress, and had clear lung sounds. Heart sounds were regular and tachycardic. The thyroid gland was palpably enlarged. There was no edema or size difference in the lower limbs. Vital signs included a blood pressure of 140/90 mmHg, heart rate of 110 bpm, respiratory rate of 18 breaths per minute, oxygen saturation of 89% on room air, and a body temperature of 37.2°C.

Initial laboratory tests revealed mild anemia and elevated D-dimer levels (Table 1). The patient underwent pulmonary CT angiography due to suspicion of pulmonary embolism, which was negative for embolism but showed bilateral diffuse alveolar involvement in the perihilar regions and enlargement of both thyroid lobes with extension to retrosternal fossa, with no evidence of pleural effusion (Fig. 1). Differential diagnoses based on history and imaging findings included hemoptysis due to acute pulmonary edema or DAH. Emergency echocardiography showed an EF of 50-55%, mild diastolic dysfunction, mild mitral regurgitation, TRG:20, and SPAP: 25 mmHg. NT-proBNP level was within normal range, effectively excluding heart failure with preserved ejection fraction (HFpEF) as a cause of the patient's symptoms. Since echocardiographic findings did not support cardiogenic pulmonary edema and NT-proBNP was normal, pulse methylprednisolone 1000 mg was initiated with suspicion of DAH, and serologic tests were ordered to determine etiology. The patient underwent flexible bronchoscopy with serial bronchoalveolar lavage, which showed increasing blood in three consecutive aliquots (Fig. 2). Due to the high-risk condition of the patient, lung biopsy was not performed. Cytologic examination revealed approximately 50% hemosiderin-laden macrophages, and lavage fluid smear and culture were negative. Thus, with a confirmed diagnosis of DAH, pulse methylprednisolone 1000 mg was continued for three days, followed by oral prednisolone 50 mg daily for two days. Urinalysis showed no microscopic hematuria, and 24-h urine collection revealed no proteinuria. Serologic tests for collagen vascular diseases were all normal. Given the enlarged thyroid gland and unexplained tachycardia, thyroid function tests were ordered, which indicated hyperthyroidism (Table 1). Considering the patient's age, the onset of thyrotoxicosis and unexplained tachycardia after the first trimester, and the palpable thyroid enlargement, Graves' disease was strongly suspected. Normal ESR, absence of fever, and no recent upper respiratory infection made thyroiditis unlikely. The level of T4 and T3 and fully suppressed TSH alongside with positive TRAb were confirmatory of true hyperthyroidism rather than pregnancy associated alterations in thyroid hormone measurement. The patient was diagnosed with DAH in the context of Graves' disease and started on methimazole 5 mg every 8 h on day six, with prednisolone discontinued. During hospitalization, the patient's oxygen saturation improved, and hemoptysis and dyspnea resolved without further intervention. At 40 weeks of gestation, she delivered a healthy baby via uncomplicated vaginal delivery. The patient was discharged in good condition but unfortunately did not follow up due to financial constraints.

**Table 1:** Laboratory datas.

Test (Unit)	Result	Normal Range
WBC count (10*3/μL)	6.9	4-10
Hemoglobin (g/dL)	9.8	14-17.5
MCV	84	80-100
Platelet (10*3/μL)	259	150-450
AST (IU/ml)	24	<37
ALT (U/L)	33	<41
ALP (U/L)	188	80-306
Bili T (mg/dL)	0.9	0.3-1.2
LDH (U/L)	357	<480
BUN (mg/dL)	16	8.8-20.5
Creatinine (mg/dL)	0.5	0.7-1.4
Sodium (mEq/L)	139	135-145
Potassium (mEq/L)	3.7	3.5-5
ESR (mm/1hr)	16	Up to 20
CRP (mg/L)	1	Up to 6
D-dimer (mg/mL DDU)	310	<243.00
NT-ProBNP (pg/ml)	180	<300
Troponin I (ng/ml)	<0.3	Up to 0.3
Urine creatinine 24h (mg/24h)	950	800-2000
Urine protein 24h (mg/24h)	108	24-141
TSH (miciu/ml)	<0.005	0.3-5
T3 (ng/ml)	3.42	0.8-2.0
T4 (nmol/L)	320	59-154
T3 Uptake (%)	40.2	25-35
TRAb (IU/L)	3.22	<1.75
RF (IU/ml)	16	Up to 20
Anti CCP Ab (U/ml)	Negative [1]	Positive>25
ANA	Negative	IF method Negative <1/40
Anti ds-DNA	Negative [7]	IF method Negative <1/10
C3 (mg/dL)	107	90-180
C4 (mg/dL)	38	10-40
CH50 (%)	110	70-150
ANCA-C (U/ml)	Negative [0.2]	Negative <10
ANCA-P(U/ml)	Negative [0.2]	Negative <5
Anti GBM Ab	Negative	Up to 1/10
Transglutaminase IgG	Negative [0.2]	Negative <0.8

**Fig. 1:**
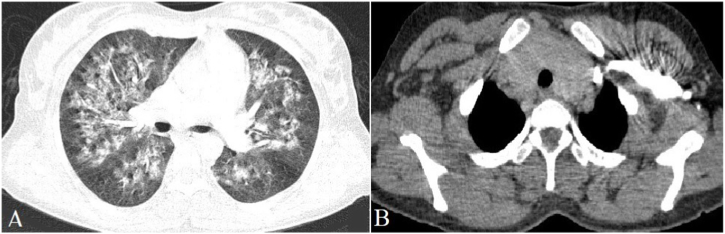
Chest CT Scan in Lung window shows Bilateral perihilar Ground-Glass Opacity and consolidation and relative spare of peripheral regions (A). Mediastinal window shows bilateral enlargement of thyroid lobes with extension into the substernal area without pressure effect on trachea (B).

**Fig. 2:**
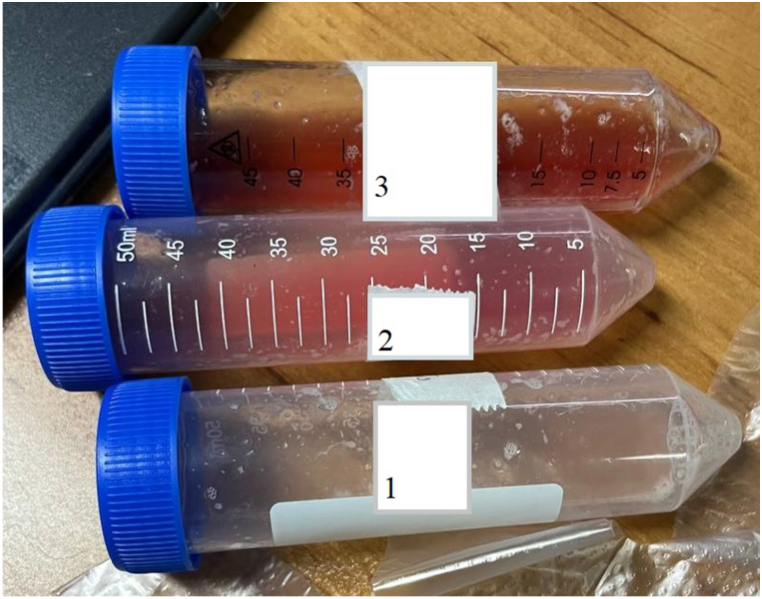
In sequential Bronchoalveolar Lavage aliquots showed progressively blood-tinged.

## 3 Discussion

DAH is a medical emergency. DAH is a rare condition, and its occurrence during pregnancy is even rarer [8]. Once DAH is diagnosed, the underlying cause must be identified. The most common causes of DAH include granulomatosis with polyangiitis, microscopic polyangiitis, isolated pulmonary capillaritis, Goodpasture syndrome, systemic lupus erythematosus, and idiopathic pulmonary hemosiderosis. The presence of proteinuria and abnormal urinary sediment (red blood cells and red blood cell casts) suggests glomerulonephritis and is consistent with systemic vasculitis, Goodpasture syndrome, connective tissue diseases, and rarely, infection [7,9,10].

When drug-induced causes, environmental exposures, mitral valve disorders, bleeding disorders, and conditions predisposing to ARDS are ruled out, four diagnoses should be considered: Goodpasture syndrome limited to the lungs, isolated pulmonary capillaritis with P-ANCA, isolated pulmonary capillaritis without autoantibodies, and idiopathic pulmonary hemosiderosis [3]. Causes of bland pulmonary hemorrhage include factors that increase left ventricular pressure and end-diastolic pressure, leading to hemorrhage [1].

The prevalence of thyrotoxicosis during pregnancy is approximately 0.2% [11]. Untreated thyrotoxicosis during pregnancy increases maternal and fetal mortality and morbidity [12]. The risk of gestational hypertension and preeclampsia in the mother increases, likely due to increased sensitivity to adrenergic activity and increased activity of the renin-angiotensin-aldosterone system [13].

The concept of peripartum heart failure with preserved ejection fraction (HFpEF) has recently emerged as a distinct clinical entity [14]. Yurista et al. [14] described a novel phenotype of peripartum HF characterized by elevated natriuretic peptides with preserved left ventricular ejection fraction, distinct from peripartum cardiomyopathy (PPCM). According to the European Society of Cardiology (ESC) diagnostic pathway, patients presenting with signs and symptoms of heart failure in the peripartum period should undergo evaluation of natriuretic peptide levels and echocardiographic assessment of left ventricular ejection fraction [14,15]. In our patient, echocardiography demonstrated an ejection fraction of 50-55% with only mild diastolic dysfunction and mild mitral regurgitation. Crucially, NT-proBNP levels were measured and found to be within normal range (<300 pg/mL). According to ESC recommendations, an NT-proBNP level below 300 pg/mL provides a high probability of ruling out heart failure [14]. The combination of normal NT-proBNP levels and preserved ejection fraction effectively excluded peripartum HFpEF as well as PPCM, which is characterized by left ventricular systolic dysfunction with LVEF below 45% [15]. Furthermore, the absence of preeclampsia, hypertension, or other risk factors for peripartum cardiac dysfunction, along with normal NT-proBNP levels, supported the conclusion that the patient's DAH was not secondary to pregnancy-related cardiac dysfunction.

In the discussed case, we propose that in the absence of coagulopathy, exposure to inhaled toxins, and no evidence of autoantibodies, untreated hyperthyroidism may have contributed to left ventricular diastolic dysfunction, exerting shear stress on the pulmonary vasculature and potentially resulting in DAH. However, it is important to acknowledge that causality cannot be definitively established from a single case report, and several alternative explanations warrant consideration.

The patient's echocardiographic findings—mildly reduced left ventricular systolic function (EF 50-55%), mild diastolic dysfunction, and mild mitral regurgitation—raise the possibility that elevated left-sided filling pressures may have contributed to the pulmonary hemorrhage. However, the normal NT-proBNP level in this patient argues against clinically significant elevation of left-sided filling pressures. In normal pregnancy, significant or pronounced elevation of B-type natriuretic peptide and NT-proBNP levels is not usually observed [15]. Therefore, the normal NT-proBNP level provides further support that the pulmonary hemorrhage was not primarily due to cardiogenic causes. Given the patient's late gestational stage, thyrotoxicosis-associated cardiac dysfunction, or high-output heart failure could theoretically contribute, but these were ruled out by the normal NT-proBNP and echocardiographic findings that did not support cardiogenic pulmonary edema. The radiographic findings of bilateral perihilar ground-glass opacity with relative sparing of peripheral regions could be compatible with pulmonary hemorrhage rather than pulmonary congestion, further supporting the diagnosis of DAH rather than cardiogenic pulmonary edema.

After reviewing the literature, several cases of DAH associated with propylthiouracil use have been reported, but only two cases of DAH in untreated Graves' disease have been found, with only one case report available [16]. This scarcity of reported cases underscores the need for further investigation into the potential pathophysiological link between Graves' disease and DAH.

Therefore, in cases of bland hemorrhage, rarer causes such as thyroid disorders should also be considered, particularly when more common etiologies have been excluded. The association between Graves' disease and DAH in this case is suggestive, but further research and additional case reports are needed to establish a definitive causal relationship.

## 4 Limitations

The main limitation of this report is that due to the high-risk condition of the patient (pregnancy and active hemorrhage), lung biopsy was not performed. While bronchoscopy and BAL provided strong evidence for DAH, a biopsy could have offered more definitive histopathological confirmation of the underlying cause. Additionally, the patient was lost to follow-up after discharge due to financial constraints. This prevented long-term monitoring of her thyroid function, pulmonary health, and potential recurrence of DAH. Follow-up data could have provided additional insights into the long-term outcomes of the treatment and the natural course of the disease.

## 5 Conclusion

DAH is an emergency and life-threatening condition, and identifying the underlying cause and appropriate treatment can be lifesaving. In cases of bland hemorrhage where no cause is found, rarer causes such as thyroid disorders should be considered. This case highlights the importance of maintaining a broad differential diagnosis and considering uncommon etiologies, particularly in pregnant patients where diagnostic evaluation may be limited by safety concerns.

## Consent to participate

Written informed consent for publication of their clinical details and clinical images was obtained from the patient.

## Consent for publication

Not applicable.

## Availability of data and materials

The datasets used during the current study are available from the corresponding author [H.A] on reasonable request.

## Clinical trial number

Not applicable.

## Funding

This research did not receive any specific grants from funding agencies in the public, commercial, or not-for-profit sectors.

## CRediT authorship contribution statement

**Sara Soltanmohammadi:** Data curation, Writing – original draft. **Hesam Amini:** Supervision, Writing – review & editing.

## Declaration of competing interest

The authors declare no competing interests.

## List of Abbreviations:

DAH: Diffuse Alveolar Hemorrhage
DAD: Diffuse Alveolar Damage
BAL: Bronchoalveolar Lavage
TSHRAb: Thyroid Stimulating Hormone Receptor Antibody
PPCM: Peripartum Cardiomyopathy
HFpEF: Heart Failure with Preserved Ejection Fraction
NT-proBNP: N-terminal pro-B-type Natriuretic Peptide
